# Supplementary material for: Factors associated with Catastrophic Healthcare Expenditure in communities of Lagos Nigeria: A Megacity experience
Source: PLoS One. 2025 Jan 24;20(1):e0316814. doi: 10.1371/journal.pone.0316814 (PMC11759386; doi:10.1371/journal.pone.0316814)
Supplement: Attachment — Submitted filename: PONE-D-24-25551_reviewer1.pdf [file pone.0316814.s001.pdf]

PLOS ONE

Factors Associated with Catastrophic Healthcare Expenditure in Communities of Lagos Nigeria: A Megacity Experience

--Manuscript Draft--

**Manuscript Number:**	PONE-D-24-25551
**Article Type:**	Research Article
**Full Title:**	Factors Associated with Catastrophic Healthcare Expenditure in Communities of Lagos Nigeria: A Megacity Experience
**Short Title:**	Catastrophic Healthcare Expenditure in Communities of Lagos, Nigeria
**Corresponding Author:**	Kikelomo Ololade Wright<br>Lagos State University College of Medicine<br>Lagos, Lagos NIGERIA
**Keywords:**	Catastrophic Health Expenditure, Prevalence, Household Experience, Health Insurance Enrollment
**Abstract:**	Background: Each year, millions of people in low—and middle-income countries such as Nigeria are forced into poverty and financial ruin due to out-of-pocket (OOP) healthcare expenses. Our study assessed the prevalence and determinants of Catastrophic Healthcare Expenditure (CHE) experienced by households in Lagos, Nigeria.<br>Methods: A descriptive community-based cross-sectional survey was conducted on 2492 households in Lagos from December 2022 to March 2023 in 4 Local Government Areas (LGAs) using a multistage sampling technique. Data was collected using pre-tested semi-structured questionnaires, and analysis was performed using STATA 15.0 software. Univariate, bivariate, and binary logistic regression analyses were conducted with statistical significance set at p<0.05, and a 95% Confidence Interval was calculated for the adjusted odds ratio (OR).<br>Results: The study revealed that 15% of households experience catastrophic health expenditure (CHE). Gender, marital status, educational level, occupation, personal income, health perception, household location, and health insurance enrollment were found to be significantly associated with CHE. Additionally, gender, religion, income, household location, and self-rated health status were identified as significant predictors of household CHE, with adjusted odds ratios of 4.42, 2.45, 1.00, 1.07, and 1.69, respectively.<br>Conclusion: CHE is more prevalent among people with lower socio-economic status, urban residents, and those lacking health insurance. It is crucial to implement targeted initiatives to raise awareness about the benefits of health insurance and simplify the enrollment process for vulnerable groups, thereby alleviating the financial strain of healthcare expenses.
**Order of Authors:**	Kikelomo Ololade Wright
	Adeyinka Adeniran
	Adedayo Aderibigbe
	Olufunsho Akinyemi
	Temiloluwa Fagbemi
	Omoyeni Ayodeji
	Biola Adepase
	Emmanuella Zamba
	Hussein Abdurrazzaq
	Faith Oniyire
	Olusegun Ogboye
	Akin Abayomi

Powered by Editorial Manager® and ProduXion Manager® from Aries Systems Corporation

Additional Information:	
Question	Response
**Financial Disclosure**<br><br>Enter a financial disclosure statement that describes the sources of funding for the work included in this submission. Review the submission guidelines for detailed requirements. View published research articles from *PLOS ONE* for specific examples.<br><br>This statement is required for submission and **will appear in the published article** if the submission is accepted. Please make sure it is accurate.<br><br>**Funded studies**<br>Enter a statement with the following details:<br>• Initials of the authors who received each award<br>• Grant numbers awarded to each author<br>• The full name of each funder<br>• URL of each funder website<br>• Did the sponsors or funders play any role in the study design, data collection and analysis, decision to publish, or preparation of the manuscript?<br><br>Did you receive funding for this work?	Yes
Please add funding details.<br>as follow-up to "**Financial Disclosure**<br><br>Enter a financial disclosure statement that describes the sources of funding for the work included in this submission. Review the submission guidelines for detailed requirements. View published research articles from *PLOS ONE* for specific examples.<br><br>This statement is required for submission and **will appear in the published article** if the submission is accepted. Please make sure it is accurate.	BILL AND MELINDA GATES FOUNDATION<br><br>INV-016366

**Funded studies**<br>Enter a statement with the following details:<br>• Initials of the authors who received each award<br>• Grant numbers awarded to each author<br>• The full name of each funder<br>• URL of each funder website<br>• Did the sponsors or funders play any role in the study design, data collection and analysis, decision to publish, or preparation of the manuscript?<br><br>Did you receive funding for this work?"	
Please select the country of your main research funder (please select carefully as in some cases this is used in fee calculation).<br>as follow-up to "**Financial Disclosure**<br><br>Enter a financial disclosure statement that describes the sources of funding for the work included in this submission. Review the submission guidelines for detailed requirements. View published research articles from *PLOS ONE* for specific examples.<br><br>This statement is required for submission and **will appear in the published article** if the submission is accepted. Please make sure it is accurate.<br><br>**Funded studies**<br>Enter a statement with the following details:<br>• Initials of the authors who received each award<br>• Grant numbers awarded to each author<br>• The full name of each funder<br>• URL of each funder website<br>• Did the sponsors or funders play any role in the study design, data collection and analysis, decision to publish, or preparation of the manuscript?<br><br>Did you receive funding for this work?"	NIGERIA - NG
**Competing Interests**	The authors have declared that no competing interests exist.

Use the instructions below to enter a competing interest statement for this submission. On behalf of all authors, disclose any competing interests that could be perceived to bias this work—acknowledging all financial support and any other relevant financial or non-financial competing interests.<br><br>This statement is required for submission and **will appear in the published article** if the submission is accepted. Please make sure it is accurate and that any funding sources listed in your Funding Information later in the submission form are also declared in your Financial Disclosure statement.<br><br>View published research articles from *PLOS ONE* for specific examples.<br><br>**NO authors have competing interests**<br><br>Enter: *The authors have declared that no competing interests exist.*<br><br>**Authors with competing interests**<br><br>Enter competing interest details beginning with this statement:<br><br>*I have read the journal's policy and the authors of this manuscript have the following competing interests: [insert competing interests here]*<br><br>* typeset	
**Ethics Statement**<br><br>Enter an ethics statement for this submission. This statement is required if the study involved:<br><br>• Human participants<br>• Human specimens or tissue<br>• Vertebrate animals or cephalopods<br>• Vertebrate embryos or tissues	Ethical approval was obtained from the Health Research Ethics Committee of the Lagos State University Teaching Hospital, Nigeria (LREC/06/10/1866). Each respondent provided written consent and was assured of the confidentiality of their information and their right to withdraw from the study at any point in time.

• Field research<br><br>Write "N/A" if the submission does not require an ethics statement.<br><br>General guidance is provided below. Consult the submission guidelines for detailed instructions. **Make sure that all information entered here is included in the Methods section of the manuscript.**	

**Format for specific study types**<br><br>**Human Subject Research (involving human participants and/or tissue)**<br>• Give the name of the institutional review board or ethics committee that approved the study<br>• Include the approval number and/or a statement indicating approval of this research<br>• Indicate the form of consent obtained (written/oral) or the reason that consent was not obtained (e.g. the data were analyzed anonymously)<br><br>**Animal Research (involving vertebrate animals, embryos or tissues)**<br>• Provide the name of the Institutional Animal Care and Use Committee (IACUC) or other relevant ethics board that reviewed the study protocol, and indicate whether they approved this research or granted a formal waiver of ethical approval<br>• Include an approval number if one was obtained<br>• If the study involved *non-human primates*, add *additional details* about animal welfare and steps taken to ameliorate suffering<br>• If anesthesia, euthanasia, or any kind of animal sacrifice is part of the study, include briefly which substances and/or methods were applied<br><br>**Field Research**<br><br>Include the following details if this study involves the collection of plant, animal, or other materials from a natural setting:<br>• Field permit number<br>• Name of the institution or relevant body that granted permission	
**Data Availability**<br><br>Authors are required to make all data underlying the findings described fully available, without restriction, and from the time of publication. PLOS allows rare exceptions to address legal and ethical concerns. See the PLOS Data Policy and FAQ for detailed information.	No - some restrictions will apply

A Data Availability Statement describing where the data can be found is required at submission. Your answers to this question constitute the Data Availability Statement and **will be published in the article**, if accepted.<br><br>**Important:** Stating 'data available on request from the author' is not sufficient. If your data are only available upon request, select 'No' for the first question and explain your exceptional situation in the text box.<br><br>Do the authors confirm that all data underlying the findings described in their manuscript are fully available without restriction?	
**Describe where the data may be found in full sentences. If you are copying our sample text, replace any instances of XXX with the appropriate details.**<br><br>• If the data are **held or will be held in a public repository**, include URLs, accession numbers or DOIs. If this information will only be available after acceptance, indicate this by ticking the box below. For example: *All XXX files are available from the XXX database (accession number(s) XXX, XXX.*).<br>• If the data are all contained **within the manuscript and/or Supporting Information files**, enter the following: *All relevant data are within the manuscript and its Supporting Information files.*<br>• If neither of these applies but you are able to provide **details of access elsewhere**, with or without limitations, please do so. For example:<br><br>*Data cannot be shared publicly because of [XXX]. Data are available from the XXX Institutional Data Access / Ethics Committee (contact via XXX) for researchers who meet the criteria for access to confidential data.*<br><br>*The data underlying the results presented in the study are available from (include the name of the third party*	Data are available from the Lagos State University College of Medicine Institutional Data Access (contact via the authors) for researchers who meet the criteria for access to confidential data.

and contact information or URL).<br>• This text is appropriate if the data are owned by a third party and authors do not have permission to share the data.<br><br>* typeset	
Additional data availability information:	

Manuscript

Click here to access/download;Manuscript;CHE Manuscript_Final.docx

Factors Associated with Catastrophic Healthcare Expenditure in Communities of Lagos Nigeria: A Megacity Experience.

Running title: Catastrophic Healthcare Expenditure in Communities of Lagos, Nigeria

*Kikelomo Ololade Wright,[1,2] Adeyinka Adeniran,[1,2] Adedayo Aderibigbe,[2] Olufunsho Akinyemi,[2] Temiloluwa Fagbemi,[3] Omoyeni Ayodeji,[3] Biola Adepase,[4] Emmanuella Zamba,[4] Hussein Abdurrazzaq,[5] Faith Oniyire,[5] Olusegun Ogboye,[5] Akin Abayomi[5]

[1] Department of Community Health and Primary Healthcare, Lagos State University College of Medicine, Nigeria.

[2] Department of Community Health and Primary Healthcare, Lagos State Teaching Hospital, Nigeria

[3] Centre for Reproductive Health Research and Innovation, Lagos State University College of Medicine, Lagos, Nigeria

[4] Lagos State Ministry of Health, Lagos, Nigeria

[5] Lagos State Health Management Agency, Lagos, Nigeria

*Corresponding Author:

kikelomo.wright@lasucom.edu.ng (KO)

ORCID ID: 0000-0002-5776-498X

Abstract

Background: Each year, millions of people in low—and middle-income countries such as Nigeria are forced into poverty and financial ruin due to out-of-pocket (OOP) healthcare expenses. Our study assessed the prevalence and determinants of Catastrophic Healthcare Expenditure (CHE) experienced by households in Lagos, Nigeria.

Methods: A descriptive community-based cross-sectional survey was conducted on 2492 households in Lagos from 5th December 2022 to 31st March 2023 in 4 Local Government Areas (LGAs) using a multistage sampling technique. Data was collected using pre-tested semi-structured questionnaires, and analysis was performed using STATA 15.0 software. Univariate, bivariate, and binary logistic regression analyses were conducted with statistical significance set at $p<0.05$, and a 95% Confidence Interval was calculated for the adjusted odds ratio (OR).

Results: The study revealed that 15% of households experience catastrophic health expenditure (CHE). Gender, marital status, educational level, occupation, personal income, health perception, household location, and health insurance enrollment were found to be significantly associated with CHE. Additionally, gender, religion, income, household location, and self-rated health status were identified as significant predictors of household CHE, with adjusted odds ratios of 4.42, 2.45, 1.00, 1.07, and 1.69, respectively.

Conclusion: CHE is more prevalent among people with lower socio-economic status, urban residents, and those lacking health insurance. It is crucial to implement targeted initiatives to raise awareness about the benefits of health insurance and simplify the enrollment process for vulnerable groups, thereby alleviating the financial strain of healthcare expenses.

Keywords: Catastrophic Health Expenditure, Prevalence, Household Experience, Health Insurance Enrollment

Introduction

Worldwide, universal health coverage (UHC) is crucial in achieving access to quality health care, a fundamental human right essential for positive health outcomes.[1]

In developing nations such as Nigeria, practically all governments spend less than the prescribed 15% of their budget on health, which is inconsistent with the Abuja Declaration.[2,3]

Each year, 100 million people are forced into poverty due to out-of-pocket OOP expenses on health services, while 150 million people experience financial ruin.[4] Also, approximately 2 billion people are currently experiencing financial difficulties, of which 1 billion are facing high OOP medical expenses that can be catastrophic. OOP accounts for 32% of each country's health expenditure.[1] For instance, although healthcare in Nigeria is funded through various sources, the primary source of healthcare financing in the country is out-of-pocket spending, with over 70% of Nigerians depending on out-of-pocket healthcare services.[5]

The World Health Organization recommends that health expenses should not exceed 10% of total household consumption or 25% of income to prevent various consequences such as catastrophic health expenditure (CHE) which generally refers to out-of-pocket (OOP) medical costs that exceed a household's financial capability level.[6] According to the World Health Organization (WHO) CHE is defined as out-of-pocket payments that exceed a certain threshold (40%) of a household’s non-subsistence income.[7,8,9]

In developed nations, several factors have been associated with catastrophic health expenses at the household level. These factors include insurance coverage, the age of household members,

the presence of chronic illness in the household, the socioeconomic status and the use of healthcare facilities. [9,10,11]

To protect its citizens from excessive healthcare expenditures, the Nigerian National Health Insurance Scheme (NHIS) was established in 1999 and introduced in 2005 to provide financial risk protection while reducing the heavy burden of OOP spending on individuals and households. This system is being implemented in various states nationwide at different levels.[12]

The implementation of health insurance in Nigeria is being impeded by several challenges, including poor health service delivery at public facilities, barriers to female education, fragmented public health insurance schemes, lack of political will, and low uptake of available health insurance, among others.[13] [1, 2] [14, 15, 16]

Studies have shown that the prevalence of catastrophic health expenditure in Nigeria varies between 2.5% and 44.0% at sub-national levels, particularly with the uneven distribution of resources across the country.[10,17]

Estimating healthcare financial spending at subnational levels is needed to implement the NHIS more efficiently and effectively, achieve wider coverage of universal healthcare, reduce CHE from OOP payments, and improve health outcomes.

As the most populous Nigerian city, the Lagos State government signed a law on establishing the Lagos State Health Management Agency (LASHMA) in 2015, to ensure that all state citizens have access to quality healthcare services without catastrophic financial outcomes.[13,18]

As part of efforts to guide decision-making, this study was conducted in Lagos to determine the prevalence of household CHE and its determinants among residents of Lagos, Nigeria

Materials and Methods

Lagos State, situated in Nigeria's southwestern coastal area, has the smallest land mass in the country at the subnational level but constitutes the nation's most densely populated state, with an estimated 21 million residents and an annual growth rate between 4% and 8%. The state

encompasses 3,577 square kilometers, resulting in a population density of approximately 16,067 individuals per square kilometer. It comprises 20 Local Government Areas (LGAs) divided into 4 rural and 16 urban LGAs with several peri-urban slums[4].

Study Design and Population

This was a descriptive community-based cross-sectional study to assess CHE and its determinants among residents of Lagos State.

The study participants include consenting individuals aged 18 years and above residing in Lagos at the time of the study.

The sample size was calculated using Fisher's formula for populations>10,000, using a standard normal deviation of 1.96, a proportion of respondents experiencing CHE in a previous study (60%), an 11 p-value of 0.05 for maximum variability, and an error margin of 0.05.

A minimum sample size of 369 was calculated for each Local Government Area (LGA) and adjusted to 554, assuming a design effect of 1.5, but increased to 1000 per LGA to further improve the power of this study. Therefore, the study used an estimated sample size 4000 for the selected 4 LGAs.

Sampling method

A multi-stage sampling technique was used for the selection process. Firstly, a simple random technique was used to select four (3 urban and 1 rural) LGAs from a sampling frame of 20 (16 urban and 4 rural) LGAs in the state by balloting. Secondly, one ward was randomly selected from each LGA. Using a sampling frame of all the streets in the selected wards, at least ten streets were chosen, and in the third stage, houses on each street were selected by systematic random sampling based on the calculated sample interval. Consenting adults in each household were approached for the study. In cases with more than one household or consenting adult, one was chosen by balloting.

Study instrument

The research tool used in this study was a pretested questionnaire that included both open and closed-ended questions. The questionnaire was divided into three sections: the first section collected information on the socio-demographic and economic characteristics of the respondents, the second section focused on the respondents' general and healthcare expenditure, and the third section gathered information on the respondents' perceived health status and OOP payment for healthcare, including health insurance enrollment. The instrument was face-validated by all the investigators, and Cronbach's alpha reliability coefficient of 0.75 was computed.

Data Collection Techniques

The data collected was efficiently managed using REDCap hosted at the Lagos State University College of Medicine (LASUCOM). The REDCap database offers a centralized platform for data management. Additionally, geo-coordinates were utilized to enhance the accuracy of the data collected, ensuring that the information was precise and reliable.

Variables

The explanatory variables include age (in years), sex, marital status, the highest level of education, income level per month (Naira), household consumption expenditure, occupation which was classified into 5 (senior professionals such as academic professionals, senior admin executives; professionals such as teachers, nurses, etc.; skilled workers such as clerks, typists, etc; semi-skilled such as artisans, traders and lastly unskilled workers), location of household, whether rural or urban, enrollment in health insurance and current health status while the outcome variable was the presence or absence of CHE.

In this study, a respondent was considered to have incurred Catastrophic Health Expenditure (CHE) if their out-of-pocket (OOP) health expenditure, as a percentage of their annual income (excluding food expenditure), exceeded 40%. The 40% threshold was selected based on its widespread use in previous studies on catastrophic expenditure. The data collected includes

healthcare expenses in Naira and the funding source for the treatment. All costs associated with healthcare visits to the hospital were documented to calculate the total health expenditure.

We estimated the annual household income by requesting details of monthly income from all income earners in the household and adding them together. Using appropriate multipliers, we estimated an average for those without a steady income and converted daily and weekly pay to monthly income. The annual income was calculated by multiplying the monthly income by 12 using the conversion rate of N780 to $1 at the time of the study.

Statistical analysis

Completed questionnaires from the REDCAP platform were cleaned and coded on Microsoft Excel 2018 and exported to STATA 15.0 software (StataCorp LLC Lakeway Drive, College Station, Texas) for analysis. Data on sociodemographic information and CHE were presented using descriptive statistics. Bivariate analysis was done to determine the association between variables and CHE using significance tests.

CHE (catastrophic health expenditure) was defined as "not catastrophic=0" and "catastrophic=1" for bivariate analysis and logistic regression. The study looked at the relationship between explanatory variables such as age, gender, occupation, income, and the presence or absence of CHE. The significance level was set at 5% and logistic regression models that accounted for the survey design were used to identify the independent predictors of CHE. The logistic regression model included all significant variables in the bivariate analysis and presented as unadjusted odds ratios.

Ethical Considerations

Ethical approval was obtained from the Health Research Ethics Committee of the Lagos State University Teaching Hospital, Nigeria (LREC/06/10/1866). Each respondent provided written consent and was assured of the confidentiality of their information and their right to withdraw from the study at any point in time.

Results

The study found that the mean age of the participants was 36.4 years (± 12.9), with 66.7% being below 40 years of age. More than half (56.5%) were married, and 68.6% had completed at least a secondary education. About one-fifth (20.6%) were non-skilled laborers, and 18.4% earned below the national minimum wage of N30,000. On average, each household had about 6 occupants, and a quarter of the respondents lived in rural areas of Lagos. (Table 1)

Table 1: Sociodemographic data of respondents

**Variables**	**Frequency (N=2492)**	**Percentage**
**Age**		
<30	859	34.5%
30-40	802	32.2%
41-50	477	19.1%
51-60	222	8.9%
>60	129	5.2%
Non-response	3	0.1
Mean ± SD	36.35 ± 12.85	
**Gender**		
Male	1260	50.6%
Female	1230	49.3%
Non-response	2	0.1%
**Marital Status**		
Single	926	37.2%
Married	1406	56.4%

Widow/Widower	121	4.9%
Others	36	1.4%
Non-response	2	0.1%
**Education Level**		
Primary	233	9.3%
Secondary	1475	59.2%
Tertiary	635	25.5%
Postgraduate	77	3.1%
No Formal Education	70	2.8%
Non-response	2	0.1%
**Occupation**		
Senior professionals,	77	3.1%
Professionals	311	12.5%
Skilled workers	731	29.3%
Semi-skilled	413	16.3%
Unskilled workers	514	20.6%
Non-response	446	17.9%
**Monthly Income (₦)**		
<30,000	458	18.4%
30,000 – 50,000	852	34.3%
50,001 – 100,000	849	34.2%
>100,000	324	13.0%
Mean ± SD	72750.11 ± 109518.35	
**Location of household**		
Rural	623	25.0%

Urban	1866	74.9%
Non-response	2	0.1%
**Number of people in the household**		
<4	1126	45.2%
4 – 6	1267	50.9%
>6	96	3.9%
Mean ± SD	6.39 ± 121.19	

Findings showed that the most common items respondents spent their income on were food, transportation and healthcare. The average monthly expenditure on these items in Nigerian Naira was ₦26,659 ($34.12), ₦12,240 ($15.69), and ₦6,274 ($8.04) for food, transportation, and healthcare, respectively. (Table 2)

Table 2: CONSUMPTION EXPENDITURE OF HOUSEHOLDS

Variables	Mean ± SD	Median (Min-Max)	25% -75%
Food	26659.97 ± 21408.93	25000.00(0.00 - 300000.00)	15000.00 - 30000.00
Education	2.31 ± 0.79	2.00(1.00 - 5.00)	2.00 - 3.00
Healthcare	6274.80 ± 13523.88	4000.00(0.00 - 450000.00)	2000.00 - 6000.00
Recreation	3531.60 ± 7815.25	0.00(0.00 - 100000.00)	0.00 - 4000.00
Transportation	12240.38 ± 16254.73	6000.00(0.00 - 300000.00)	2800.00 - 15000.00
Communication	5721.99 ± 8474.54	4000.00(0.00 - 250000.00)	2000.00 - 6000.00

Cloths	5322.25 ± 9327.68	3000.00(0.00 - 150000.00)	0.00 - 6000.00
Smoking	549.85 ± 5312.05	0.00(0.00 - 250000.00)	0.00 – 0.00
Alcohol	2997.97 ± 7822.18	0.00(0.00 - 100000.00)	0.00 - 2000.00

One-third of the respondents believed their health status was excellent, 41.0% said it was very good, while less than 1.0% rated their health status as poor. Hypertension accounted for 49.2% of all reported morbidity, while 36.0% of respondents were aware of health insurance, with 30% enrolled in any scheme. The average monthly healthcare spending was ₦6,221.38 ($7.78), funded primarily through cash savings. (Table 3)

Table 3: RESPONDENTS' HEALTH STATUS AND THEIR PERCEPTION

**VARIABLE**	**Freq. (n=2492)**	**Percentage**
**Respondents' rating of current health status**		
Excellent	804	32.3%
Very good	1022	41.0%
Good	571	22.9%
Fair	86	3.5%
Poor	6	0.2%
Non-response	3	0.1%
**Any morbidity (known health condition)**		
Yes	313	12.6%
No	2093	84.0%
Don't know	83	3.3%

Non-response	3	0.1%
**Morbidity type**		
Diabetes	86	27.5%
Hypertension	154	49.2%
Others*	73	23.3%
**Recent illness in past 4 weeks (Acute)**		
Yes	332	13.3%
No	2154	86.4%
Non-response	6	0.2%
**Respondent/ immediate family received care from health provider in the last 4 weeks.**		
Yes	577	23.2%
No	1910	76.6%
Non-response	5	0.2%
**Illness duration (days)**		
Mean ± SD	3.52 ± 3.34	
**Amount spent on treatment and services (Naira)**		
Mean ± SD	10282.00 ± 23035.72	
**Estimated monthly household spending on healthcare (Naira)**		
Mean ± SD	6221.38 ± 9456.61	
**Source(s) of money to meet costs of treatment (coping with cost)**		
Cash savings	1917	77.1%
Borrowing	145	5.8%
Gifts	139	5.6%
Credit	30	1.2%

Sell assets	4	0.2%
Health Insurance	187	7.5%
Others	65	2.6%
**Hospitalization (at least overnight stay in the last 6 months)**		
Yes	307	12.3%
No	2179	87.4%
Non-response	6	0.2%
**Ever enrolled in Health Insurance Scheme**		
Yes	270	10.8%
No	2222	89.2%
**Household spending on healthcare in last 12 months – (Naira)**		
Mean ± SD	29638.06 ± 53471.70	
Median (Min-Max)	15000(0.00 - 100000)	
25% -75%	6000.00 - 30000.00	

*Others include renal, musculoskeletal, respiratory diseases, etc.

Only 15% of the participants experienced CHE in their households. (Figure 1)

FIGURE 1: The prevalence of catastrophic health spending within households in Lagos

Gender, marital status, educational level, occupation, personal income, perception of current health status, location of a household, and enrolment in health insurance schemes were significantly associated with CHE. Females (18.7%) experienced more CHE compared to males (11.4%), and married individuals (17.5%) experienced more CHE compared to singles

(11.2%) (P<0.05). Semi-skilled respondents (19.4%) experienced more CHE than skilled respondents (16.3%) or senior professionals (10.5%) while respondents with personal income less than ₦30,000 ($38.5) experienced higher CHE (36.5%) compared to those who earned higher. Additionally, the household's location and health insurance enrollment were significantly associated with CHE. A higher proportion of respondents in the urban areas (17.6%) were more likely to experience CHE compared to those in the rural areas (7.1%), (P<0.05). Also, a higher proportion of households not enrolled in any health insurance schemes (14.1%) were more likely to experience CHE compared to those who were enrolled (7.4%) (P<0.05). Respondents' perceptions of current health status were not significantly associated with CHE (P>0.05) (Table 4)

Table 4: Relationship between CHE and sociodemographic characteristics of respondents

**Sociodemographic Variable**	**Non-CHE**	**CHE**	**Statistic**	**P-value**
**AGE**				
<30	749(87.2)	110(12.8)	$X^2 = 9.2$	0.056
30-40	678(84.6)	123(15.4)		
41-50	404(84.7)	73(15.3)		
51-60	176(79.3)	46(20.7)		
>60	108(83.7)	21(16.3)		
**Gender**				
Male	1116(88.6)	143(11.4)	$X^2 = 26.3$	<0.000**
Female	1000(81.3)	230(18.7)		
**Marital Status**				
Single	822(88.8)	104(11.2)	$X^2 = 17.7$	0.001*
Married	1159(82.5)	246(17.5)		

Widow/Widower	102(84.3)	19(15.7)		
Others	32(88.9)	4(11.1)		
**Education Level**				
Primary	191(82.0)	42(18.0)	X^2 = 17.6	0.002*
Secondary	1231(83.5)	244(16.5)		
Tertiary	572(90.1)	63(9.9)		
Postgraduate	63(82.9)	13(17.1)		
No Formal Education	59(84.3)	11(15.7)		
**Occupation**				
Senior professionals	68(89.5)	8(10.5)	X^2 = 16.9	0.002*
Professionals	283(91.0)	28(9.0)		
Skilled workers	612(83.7)	119(16.3)		
Semi-skilled workers	333(80.6)	80(19.4)		
Unskilled workers	438(85.2)	76(14.8)		
**Monthly Income (₦)**				
<30,000	290(63.5)	167(36.5)	X^2 = 239.2	<0.000*
30,000 – 50,000	726(85.2)	126(14.8)		
50,001 – 100,000	780(91.9)	69(8.1)		
>100,000	317(97.5)	8(2.5)		
**Location of household**				
Rural	579(92.9)	44 (7.1)	X^2 = 41.0	<0.000**
Urban	1536(82.4)	329 (17.6)		
**Rating current health status**				
Excellent	690(85.9)	113(14.1)	X^2 = 7.0	0.135

Very good	873(85.4)	149(14.6)		
Good	482(84.4)	89(15.6)		
Fair	66(76.7)	20(23.3)		
Poor	4(66.7)	2(33.3)		
**Known health condition**				
Yes	268(85.6)	45(14.4)	X^2 = 0.3	0.847
No	1775(84.8)	317(15.2)		
Don't know	72(86.7)	11(13.3)		
**Ever enrolled in health insurance scheme**				
Yes	250(92.6)	20(7.4)	X^2 = 8.0	0.003**
No	542(85.9)	89(14.1)		

"*" means significant P-value while "**" means Fisher's exact value

The study found that gender, religion, income, household location, and respondents' rating of health morbidity were significant predictors associated with household CHE with an adjusted odds ratio (AOR) of 4.42, 2.45, 1.00, 1.07, and 1.69, respectively, at p<0.001. (Table 5)

Table 5: CHE predictors among respondents

Variables	Adjusted OR	95% CI	P-value
Age	1.01	0.99 – 1.04	0.204
Gender	4.42	2.12 – 9.21	<0.001*
Religion	2.45	1.56 – 3.86	<0.001*
Employment	0.67	0.39 – 1.13	0.129
Education	1.08	0.79 – 1.47	0.644
Income Grp,	1.00	1.00 – 1.00	<0.001*

Occupation	1.17	0.91 – 1.51	0.221
Location of household	1.07	1.01 – 1.14	0.028*
Rate of health morbidity	1.69	1.25 – 2.29	0.001*
Head of household	3.01	0.22 – 42.02	0.413
Education of head	0.78	0.60 – 1.03	0.077
Constant	0.03	0.002 – 0.27	0.002*

* 5% significance level

Discussion

The consequences of CHE in Lagos can lead to not only poor health outcomes but also a decline in the socio-economic status of families and households that are on the verge of poverty. In this study, the average monthly expenditure on food items was ₦26,659 ($34.12) and this was similar to a previous conducted in Lagos state, which reported an average food expenditure of ₦29,282 ($37.54)[11]

At a 40% threshold of non-food expenditure, a CHE prevalence of 15% was found in our study, similar to figures reported in other studies conducted in other low-socioeconomic countries such as Burkina Faso (10.8%) and Kenya (9.8%).[20,21] Another Nigerian study also reported catastrophic health spending of 13.7%.[22] Our study has a higher CHE which is in contrast to another Kenyan study that reported a prevalence of less than 1.52 which might be due to the high incidence of OOP payment.[23]

Recent reports indicate that there has been an increase in healthcare expenditure (CHE) patterns in sub-Saharan Africa. This trend is mainly due to factors such as the higher cost of living, rising healthcare costs, a growing number of chronic ailments coupled with prevalent infecti[illegible] diseases, and the recent COVID-19 pandemic which may have further amplified the vulnerability of health systems.[24]

Our study revealed that there is a difference in the prevalence of CHE between urban and rural households in Lagos. The study found that households in urban areas had a higher prevalence of CHE (17.6%) than those in rural areas (7.1%). This finding differs from the findings of two other studies conducted in Ekiti and Ibadan, also located in southwest Nigeria, which reported a higher prevalence of CHE in rural areas than in urban settings.[19,25,26] Urban Lagos is a densely populated metropolis that serves as the economic hub of the country. The high population density has led to a correspondingly high cost of living, making the healthcare system overburdened. Consequently, the cost of healthcare services in urban areas is likely to be higher than their rural counterparts.

The study findings indicate that there is an association between CHE and various sociodemographic and socioeconomic factors such as age, gender, educational level, occupation, and income. It was observed that individuals belonging to households with lower levels of education and income experienced higher levels of CHE. Moreover, people with better socioeconomic status may have better healthcare-seeking behavior and can afford healthcare services as observed from findings in previous studies.[22,27,28] Approximately 35% of the participants in the study were under the age of 30 and had lower CHE. This age group is less likely to have chronic health conditions that require frequent healthcare visits and spending, which could be the reason for the lower CHE in this group.

The socioeconomic inequalities related to CHE can be reduced through financial risk protection of health insurance schemes. However, less than 10% of respondents who experienced CHE were enrolled in any health insurance scheme that could have offered protection. This study revealed that health insurance status has a statistically significant association with catastrophic health expenditure among respondents, contrasting with the findings from a study in Iran.[11,29] This observation confirmed that health insurance protects against CHE.

Respondents who rated their current health status as poor experienced catastrophic health expenditure more than those who rated their health as fair or excellent, though there is no statistically significant association. Those with poor health status may have higher healthcare costs similar to a study done in Lagos among persons living with HIV (PLHIV).[11]

In consonance with other studies, our findings showed that factors such as gender, religion, income, location of household and the perception of health morbidity predict the presence of CHE.[11,15,25,30]

Conclusion

Our study has shown that CHE is more common among individuals living in urban areas, those with low socio-economic status, and those without health insurance. To mitigate the financial burden of healthcare costs, it is essential to raise awareness of the benefits of health insurance and actively encourage enrolment among vulnerable groups, particularly those in lower economic strata, in both formal and informal sectors. This collective effort can effectively reduce out-of-pocket expenses and prevent the negative impact of CHE on individuals and families.

Strengths and limitations of the study:

The community-based nature and the large sample size are considered to be the major strengths that enhance the quality and robustness of our study findings.

Recall bias was minimized by targeting questions about illness and care 4 weeks before the interview. Social desirability bias was also a limitation, which was reduced by making respondents self-report on questions on household income.

Implication for policy and future research:

Increasing awareness and promoting enrollment in health insurance schemes, especially among vulnerable groups like those with low socioeconomic status and those living in urban areas can help reduce CHE from OOP payments. Also, addressing the socioeconomic inequalities that

contribute to higher CHE, such as improving access to education and employment opportunities, which can increase household income levels; strengthening the implementation and coverage of the National Health Insurance Scheme (NHIS) and the Lagos State Health Management Agency (LASHMA) health insurance programs to provide better financial risk protection to households; exploring ways to subsidize or provide financial assistance for healthcare costs to households below a certain income threshold to prevent catastrophic expenditures and impoverishment due to medical expenses are some of the policy implications of this study. Further research at subnational or national levels will be valuable in better understanding the determinants and patterns of CHE and evaluating targeted policy interventions.

Availability of data and materials: The datasets generated and/or analyzed during the current study are not publicly available and are available from the corresponding author upon reasonable request.

Competing interests: All authors have no relevant financial or non-financial interests to disclose.

Funding: Bill and Melinda Gates Foundation

Authors' Contributions:

Conception - **KOW, AA, TF, HA, EZ, OO, Ak.Ab**

Design of the work - **KOW, AA, AA, TF, BA, EZ, HA**

Data acquisition - **KOW, AA, AA, TF, OA, BA, HA, FO, OO, Ak.Ab**

Analysis and interpretation of data - **KOW, AA, AA2, OA, TF, FO**

Drafted the work or substantively revised: **KOW, AA, OA, FO**

“The funders had no role in study design, data collection and analysis, decision to publish, or preparation of the manuscript.”

References

1. Universal Health Coverage (UHC) [Internet]. [Cited 2023 Dec 13]. Available From: Https://Www.Who.Int/News-Room/Fact-Sheets/Detail/Universal-Health-Coverage-(Uhc)
2. The Abuja Declaration: Ten Years On 2001 Promises of Commitment and Solidarity. 2010 [Cited 2023 Dec 19]; Available From: Http://Www.Oecd.Org/Document/11/0,3746,En_2649_34447_44981579_1_1_1_1,00.Html
3. The Development Research and Project Centre. 20th Anniversary of The Abuja Declaration (2001-2021) Assessing Nigeria's Two-Decades Spending on The Health Sector Why Poor Allocation, Releases & Utilization Persist? – Drpc [Internet]. [Cited 2024 Apr 8]. Available From: Https://Drpcngr.Org/20th-Anniversary-Of-The-Abuja-Declaration-2001-2021-Assessing-Nigerias-Two-Decades-Spending-On-The-Health-Sector-Why-Poor-Allocation-Releases-Utilization-Persist/
4. Dave-Agboola IO, Raji JI. Health-Seeking Behaviour of Malaria Patients in Lagos, Nigeria. International Journal of Health Sciences & Research (www.Ijhsr.Org) [Internet]. 2018 [Cited 2024 May 22];8(7):259. Available From: www.Ijhsr.Org
5. Erinoso O, Oyapero A, Familoye O, Omosun A, Adeniran A, Kuyinu Y. Predictors of Health Insurance Uptake among Residents of Lagos, Nigeria. Popul Med [Internet]. 2023 Jul 31 [Cited 2024 Jun 3];5(July):1–7. Available From:

Https://Www.Populationmedicine.Eu/Predictors-Of-Health-Insurance-Uptake-Among-Residents-Of-Lagos-Nigeria,169666,0,2.Html

6. Zhang X, Xu Q, Guo X, Jing Z, Sun L, Li J, Et Al. Catastrophic Health Expenditure: A Comparative Study Between Hypertensive Patients with and without Complication in Rural Shandong, China. BMC Public Health. 2020 Apr 22;20(1).

7. SDG 3.8.2 Catastrophic Health Spending (And Related Indicators) [Internet]. [Cited 2023 Dec 13]. Available From: Https://Www.Who.Int/Data/Gho/Data/Themes/Topics/Financial-Protection/GHO/Financial-Protection

8. The World Health Report: Health Systems Financing: The Path to Universal Coverage [Internet]. [Cited 2023 Dec 20]. Available From: Https://Iris.Who.Int/Handle/10665/44371

9. Universal Health Coverage | WHO | Regional Office for Africa [Internet]. [Cited 2023 Dec 13]. Available From: Https://Www.Afro.Who.Int/Health-Topics/Universal-Health-Coverage

10. Ukwaja KN, Alobu I, Abimbola S, Hopewell PC. Household Catastrophic Payments for Tuberculosis Care in Nigeria: Incidence, Determinants, and Policy Implications for Universal Health Coverage. Infect Dis Poverty [Internet]. 2013 Sep 17 [Cited 2023 Dec 18];2(1):21. Available From: /Pmc/Articles/PMC3848689/

11. Adeniran A, Ojo OY, Chieme FC, Shogbamimu Y, Olowofeso HO, Sidibé I, Et Al. Investigating Catastrophic Health Expenditure among People Living with HIV and AIDs in South Western Nigeria. Health Care Science. 2023;

12. Ukwaja KN, Alobu I, Nweke CO, Onyenwe EC. Healthcare-Seeking Behavior, Treatment Delays and its Determinants among Pulmonary Tuberculosis Patients in

Rural Nigeria: A Cross-Sectional Study. BMC Health Serv Res [Internet]. 2013 [Cited 2024 Apr 16]; 13:25. Available From: Https://Pubmed.Ncbi.Nlm.Nih.Gov/23327613/

13. Lagos State Health Insurance Scheme - Ministry of Health [Internet]. [Cited 2023 Dec 20]. Available From: Https://Lagosministryofhealth.Org/Lagos-State-Health-Insurance-Scheme/

14. National Health Insurance [Internet]. [Cited 2023 Dec 14]. Available From: Https://Www.Nhis.Gov.Ng/

15. Onwujekwe O, Ezumah N, Mbachu C, Obi F, Ichoku H, Uzochukwu B, Et Al. Exploring Effectiveness of Different Health Financing Mechanisms in Nigeria; What Needs to Change and How Can It Happen? BMC Health Serv Res [Internet]. 2019 Dec 1 [Cited 2023 Dec 14];19(1):1–13. Available From: Https://Bmchealthservres.Biomedcentral.Com/Articles/10.1186/S12913-019-4512-4

16. Onoka CA, Hanson K, Mills A. Growth of Health Maintenance Organisations in Nigeria and The Potential for a Role in Promoting Universal Coverage Efforts. Soc Sci Med [Internet]. 2016 Aug 1 [Cited 2023 Dec 21]; 162:11–20. Available From: Https://Pubmed.Ncbi.Nlm.Nih.Gov/27322911/

17. Ilesanmi OS, Adebiyi AO, Fatiregun AA. National Health Insurance Scheme: How Protected are Households in Oyo State, Nigeria from Catastrophic Health Expenditure? Int J Health Policy Manag [Internet]. 2014 May 1 [Cited 2023 Dec 14];2(4):175–80. Available From: Https://Www.Ijhpm.Com/Article_2837.Html

18. LASHMA - Lagos State Health Scheme [Internet]. [Cited 2024 Jun 3]. Available From: Https://Www.Lashmaregulations.Com.Ng/

19. Obembe TA, Levin J, Fonn S. Prevalence and Factors Associated with Catastrophic Health Expenditure Among Slum and Non-Slum Dwellers Undergoing Emergency Surgery in a Metropolitan Area of South Western Nigeria. Plos One [Internet]. 2021

Aug 1 [Cited 2023 Dec 19];16(8): E0255354. Available From: Https://Journals.Plos.Org/Plosone/Article?Id=10.1371/Journal.Pone.0255354

20. Su TT, Kouyaté B, Flessa S. Catastrophic Household Expenditure for Health Care in a Low-Income Society: A Study from Nouna District, Burkina Faso. Bull World Health Organ [Internet]. 2006 Jan [Cited 2024 Apr 9];84(1):21. Available From: /Pmc/Articles/PMC2626518/?Report=Abstract

21. Kimani DN, Mugo MG, Kioko UM. Catastrophic Health Expenditures and Impoverishment in Kenya. European Scientific Journal, ESJ [Internet]. 2016 May 30 [Cited 2024 Apr 9];12(15):434–434. Available From: Https://Eujournal.Org/Index.Php/Esj/Article/View/7518

22. Aregbeshola BS, Khan SM. Determinants of Catastrophic Health Expenditure in Nigeria. The European Journal of Health Economics [Internet]. 2018 May 29 [Cited 2024 Feb 2];19(4):521–32. Available From: Http://Link.Springer.Com/10.1007/S10198-017-0899-1

23. Buigut S, Ettarh R, Amendah DD. Catastrophic Health Expenditure and its Determinants in Kenya Slum Communities. Int J Equity Health. 2015 May 14;14(1).

24. Eze P, Lawani LO, Agu UJ, Acharya Y. Catastrophic Health Expenditure in Sub-Saharan Africa: Systematic Review and Meta-Analysis. Bull World Health Organ [Internet]. 2022 May 1 [Cited 2024 Apr 9];100(5):337-351J. Available From: Https://Pubmed.Ncbi.Nlm.Nih.Gov/35521041/

25. Ajayi Paul Oladapo, Esan Deborah Tolulope, Ipinnimo Tope, Solomon Oluremi Olayinka. Determinants of Catastrophic Health Spending among Rural Households in Nigeria [Internet]. The Nigerian Health Journal. 2022 [Cited 2023 Dec 14]. P. 278–87. Available From:

Https://Www.Researchgate.Net/Publication/365159913_Determinants_Of_Catastrophic_Health_Spending_Among_Rural_Households_In_Nigeria

26. Ajayi PO, Ibirongbe DO, Ipinnimo TM, Solomon OO, Ibikunle AI, Obiagwu AE. The Prevalence of Household Catastrophic Health Expenditure in Nigeria: A Rural-Urban Comparison. Journal Of Health and Medical Sciences. 2021 Jun 30;4(2).
27. Ghorbanian A, Rashidian A, Lankarani KB, Kavosi Z. The Prevalence and Determinants of Catastrophic Health Expenditures in Iran: A Systematic Review and Meta-Analysis. Health Scope 2019 8:1 [Internet]. 2019 Oct 29 [Cited 2024 Mar 10];8(1):63210. Available From: Https://Brieflands.Com/Articles/Healthscope-63210
28. Ghimire M, Ayer R, Kondo M. Cumulative Incidence, Distribution, And Determinants of Catastrophic Health Expenditure in Nepal: Results from the Living Standards Survey. Int J Equity Health [Internet]. 2018 Feb 14 [Cited 2024 Mar 10];17(1). Available From: /Pmc/Articles/PMC5813388/
29. Piroozi B, Moradi G, Nouri B, Bolbanabad AM, Safari H. Catastrophic Health Expenditure After the Implementation of Health Sector Evolution Plan: A Case Study in The West of Iran. Int J Health Policy Manag. 2016;5(7):417–23.
30. Zhao Y, Oldenburg B, Mahal A, Lin Y, Tang S, Liu X. Trends and Socio-Economic Disparities In Catastrophic Health Expenditure and Health Impoverishment in China: 2010 To 2016. Tropical Medicine and International Health. 2020 Feb 1;25(2):236–47.

Figure Click here to access/download;Figure;FIGURE 1_CHE Manuscript.pdf

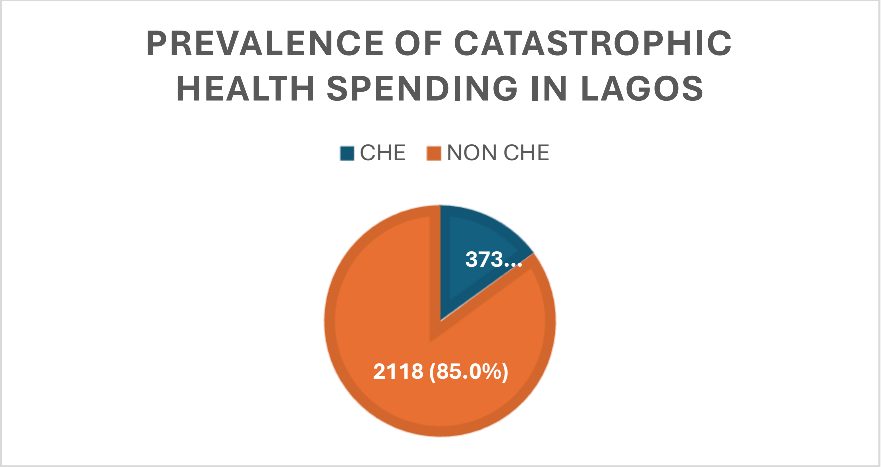

FIGURE 1: The Prevalence of Catastrophic Health Spending within Households in Lagos
