# Supplementary material for: Factors associated with Catastrophic Healthcare Expenditure in communities of Lagos Nigeria: A Megacity experience
Source: PLoS One. 2025 Jan 24;20(1):e0316814. doi: 10.1371/journal.pone.0316814 (PMC11759386; doi:10.1371/journal.pone.0316814)
Supplement: Attachment — Submitted filename: Response to Reviewers - PLOS ONE.docx [file pone.0316814.s002.docx]

**Point-by-point responses to reviewers' comments.**

Reviewer #1:

Title – Factors Associated with Catastrophic Healthcare Expenditure in Communities of Lagos
Nigeria: A Megacity Experience
- Appropriate
Abstract – Well presented.
Introduction – Statement of problem, magnitude of problem, rationale for the study and study objectives well presented.
Methods and analysis – Clearly presented. Study area description, study population, sample size calculation, sampling technique, data collection, variables and data analysis are well presented.
Study setting, target population, sampling techniques and data management are well described. Thank you

Response To Comments
1. •There are too many 1 - sentence paragraphs. Avoid 1 - sentence paragraphs
Result:
Table 4 – replace < 0.000 with < 0.001

This has been done

Discussion – Findings well discussed.

Thank you

2. Line 231 – The CHE cannot be regarded as similar (Over 50% higher). It is better referred to as "higher."

This has been corrected to read ‘’which is slightly higher than figures’’ in line 243

3. Line 235 – Compare the OOP in Kenya with Nigeria before this statement can be affirmed.

This has been corrected to read, “Our study shows a higher CHE than another Kenyan study, which indicated a prevalence of 1.55% at a 30% threshold using the WHO model and potentially a lower prevalence of under 1.52% at a 40% threshold. This difference may be explained by the monthly OOP payments of ₦6,221.38 in our study compared to the average OOP health spending of 337.7 Kenyan Shillings (approximately ₦4,269.99) in the Kenyan study. ^23^” Lines 245-250

Conclusion and Recommendations are appropriate. Thank you
References – Adequate. Thank you

4. Data Availability statement

The datasets generated and analyzed during the current study are not publicly available because they contain potentially identifying or sensitive respondents’ information, but the Lagos State University Teaching Hospital—Health Research and Ethics Committee (LASUTH-HREC) can facilitate access upon reasonable request. (Email: dcst@lasuth.org) Lines 325-328

5. Role the funders took in the study.

The funders had no role in study design, data collection and analysis, decision to publish, or preparation of the manuscript. Lines 343-344

Reviewer #2:  Thank you

1. The manuscript is technically sound.
2.. The choices of the statistical tools were appropriate for the Questions
3. All data was made available
4. The English language was presented correctly.
5. The article should be accepted. Thank you
