# Supplementary material for: Factors associated with Catastrophic Healthcare Expenditure in communities of Lagos Nigeria: A Megacity experience
Source: PLoS One. 2025 Jan 24;20(1):e0316814. doi: 10.1371/journal.pone.0316814 (PMC11759386; doi:10.1371/journal.pone.0316814)
Supplement: Attachment — Submitted filename: Response to Reviewers 12.12.2024.docx [file pone.0316814.s004.docx]

Point-by-point responses to reviewers' comments.

Introduction:

Will be appropriate if the authors can provide insight about the status of health insurance in the study state including coverage, distribution and progress made by LASHMA.

Lines 78-87

Under this scheme, state governments are also permitted to establish state-based health Insurance schemes. In 2007, Lagos State, often described as the commercial capital of Nigeria launched Community Based Health Insurance schemes to provide social health protection coverage for the poor and underserved communities while the Lagos State Health Management Agency (LASHMA) was established to provide quality healthcare and reduce out-of-pocket spending. Subsequently, LASHMA launched the Lagos State Health Insurance Scheme in 2019. A recent study conducted in Lagos revealed that only about 10.9% of respondents have ever been enrolled in any type of health insurance. Among these, private health insurance schemes were the most popular, utilized by 58.5% of participants, while less than one-fifth (15.9%) opted for the Lagos State-owned scheme. 13

Methods:

Sampling methods:

Will be good to provide a table illustrating the numbers at each stage of the selection LGAs, wards, streets etc.

 Lines 131-132

| Stage | Selection Criteria | Total Available | Selected | Selection Method |
| --- | --- | --- | --- | --- |
| Stage 1: LGAs | Urban LGAs | 16 | 3 | Simple random sampling (balloting) |
|  | Rural LGAs | 4 | 1 | Simple random sampling (balloting) |
| Stage 2: Wards | Wards within selected LGAs | Variable | 1 per LGA | Simple random sampling |
| Stage 3: Streets | Streets within selected wards | Variable | At least 10 per ward | Systematic random sampling |
| Stage 4: Houses | Houses on selected streets | Variable | Based on sampling interval of 2 | Systematic random sampling |
| Stage 5: Individuals | Consenting adults in households | Variable | 1 per household | Random selection (balloting) |

Lines 153 – 154: please indicate the year of the conversion rate. Also indicate that it is US Dollars.

DONE

Data collection:

Please indicate the periods when the data was collected, who collected the data and how the data was transmitted into REDCAP. Also highlight how data confidentiality and security was enhanced. Remember, the data was sensitive and was not shared in this submission which implies that it requires protection and security.

DONE (Lines 143-148)

Results:

Figure 1 may not be necessary since it does not add any additional value. The findings presented in the figure have been outlined in the results. Also there are only 2 variables. However, if the authors decided to retain the figure, then clearly present the caption and define the abbreviations.

FIGURE 1 HAS BEEN REMOVED

Acknowledgement:

Acknowledgements (Lines 327-329)

Please include this section at the end of the paper.

Additional reference:

13. Adeniran A, Wright K, Aderibigbe A, Akinyemi O, Fagbemi T, Ayodeji O, Adepase A, Zamba E, Abdur-Razzaq H, Oniyire F, Ogboye O, Abayomi A. Determinants of health insurance adoption among residents of Lagos, Nigeria: A cross-sectional survey. *Open Health*. 2024;5(1): 20230043. <https://doi.org/10.1515/ohe-2023-0043>
